# Cell adhesion manipulation through single cell assembly for characterization of initial cell-to-cell interaction

**DOI:** 10.1186/s12938-015-0109-2

**Published:** 2015-12-10

**Authors:** Xue Gou, Ran Wang, Stephen S. Y. Lam, Jundi Hou, Anskar Y. H. Leung, Dong Sun

**Affiliations:** Department of Mechanical and Biomedical Engineering, City University of Hong Kong, Hong Kong, China; Department of Medicine, The University of Hong Kong, Hong Kong, China

**Keywords:** Cell adhesion, Cell-to-cell interaction, Optical tweezers manipulation, Cell assembly, Leukemia-stromal cell interactions

## Abstract

**Background:**

Cell-to-cell interactions are complex processes that involve physical interactions, chemical binding, and biological signaling pathways. Identification of the functions of special signaling pathway in cell-to-cell interaction from the very first contact will help characterize the mechanism underlying the interaction and advance new drug discovery.

**Methods:**

This paper reported a case study of characterizing initial interaction between leukemia cancer cells and bone marrow stromal cells, through the use of an optical tweezers-based cell manipulation tool. Optical traps were used to assemble leukemia cells at different positions of the stromal cell layer and enable their interactions by applying a small trapping force to maintain the cell contact for a few minutes. Specific drug was used to inhibit the binding of molecules during receptor-ligand-mediated adhesion.

**Results and conclusions:**

Our results showed that the amount of adhesion molecule could affect cell adhesion during the first few minutes contact. We also found that leukemia cancer cells could migrate on the stromal cell layer, which was dependent on the adhesion state and activation triggered by specific chemokine. The reported approaches provided a new opportunity to investigate cell-to-cell interaction through single cell adhesion manipulation.

## Background

Among multiple cell behaviors, cell-to-cell interaction has received increasing attention because they provide rich information about tumor survival and metastasis [1–4]. This complex process is driven by the coordinated action of adhesion molecules anchored in the cell membrane and exchange of diffusible factors between cells [5–8]. Understanding the fundamental principles in this process is of great importance for the development of new therapeutic strategies. Traditional adhesion and transwell assays have been used to identify the effects of adhesion molecules and chemokine in cell interaction. These assays extract average information from a large number of cells, but fail to obtain the cell information at single cell level. To have in-depth understanding of the cell-to-cell interaction, elucidation of the cell adhesion through cytokines and adhesion molecules usually plays a key role. For example, to study the interaction between leukemia cells and stromal cells in bone marrow microenvironment, SDF-1/CXCR4 were found to connect the stromal cell and leukemia cell and involved in cancer therapy [9, 10]. Therefore, an efficient tool that can manipulate and control single cells for probing the functional mechanism of adhesion molecules and chemokines is highly needed.

Several new techniques have been developed to study cell-to-cell interaction processes. Atomic force microscopy (AFM) has been developed to measure cell–cell adhesion force [11]; microfluidic technology has been introduced to design co-culture systems for characterization of cell–cell interaction [12]; optical tweezers has been used to investigate the adhesion strength in receptor-ligand interaction [13], and has functioned as a force probe to observe the formation and maturation of cell adhesion [14]. Among these methods, optical tweezers exhibits the advantage of noninvasive manipulation and precise control of individual cells. Our early works have reported the use of optical tweezers combined with fluorescence microscopy technology to study cell-to-cell interaction via single cell adhesion manipulation [15, 16].

This paper presents the use of an optical tweezers-based cell manipulation tool to control cell adhesion through assembling single cells for probing initial cell-to-cell interaction. The tool is applied to a specific study on the interaction between leukemia cancer cells and stromal cells. The optical force exerted on a cell was first calibrated, and the adhesion state of leukemia cells on stromal cells was then characterized based on varied force manipulation. To investigate the influence of adhesion molecule on the interaction, the leukemia cells were assembled at different sites of the stromal cell layer by optical tweezers, which applied small trapping force to maintain the cell contact for a few minutes. The functions of chemokine in cell-to-cell interaction were studied using specific drug to block a signaling pathway involved in these processes. In a case study, the role of stroma-secreted chemokine stromal-derived factor 1 (SDF-1) and its cognate receptor CXCR4 in leukemia/bone marrow cell interaction were particularly investigated. Our findings are that adhesion molecules can largely affect the adhesion of leukemia cells on stromal cells, and leukemia cells can be induced to migrate on stromal cell layer, depending on tight adhesion and activation triggered by SDF-1.

The main contributions of this paper lie in the development of a single cell manipulation tool with optical tweezers to manipulate direct cell-to-cell contact adhesion, and application of this tool to characterize the SDF-1/CXCR4 mediated cell adhesion and migration at single cell level. The proposed approach will offer a new avenue to characterize and control cell adhesion in probing the mechanism of cell-to-cell interactions.

## Methods

### Manipulation setup and trapping force calibration

An optical tweezers cell manipulation system was established in our laboratory [17–20]. The system, with an intuitive user interface, can be used to manipulate cells at micro/nano level precision. The dichroic mirror reflects the laser beam into the objective, capturing the images of cells through a charge-coupled device camera. The positions of the cells were detected by digital image processing. An incubator was mounted on the motorized stage to keep cells in an atmosphere of 5 % CO_2_ at 37 °C.

The optical trapping force imposed on a cell at a given laser power can be calibrated based on viscous-drag-force calibration method [20]. A cell was captured in a trap at a constant separation distance *h* from the bottom of the Petri dish used in the experiments. As the whole dish was driven at a certain velocity via the motorized stage, the fluid flow exerted a viscous drag force on the trapped cell. The flow velocity increased until the cell escaped from the optical trap. With the escape velocity, the maximal trapping force at a given laser power can be calculated using the Stokes relation [21].

**Fig. 1:**
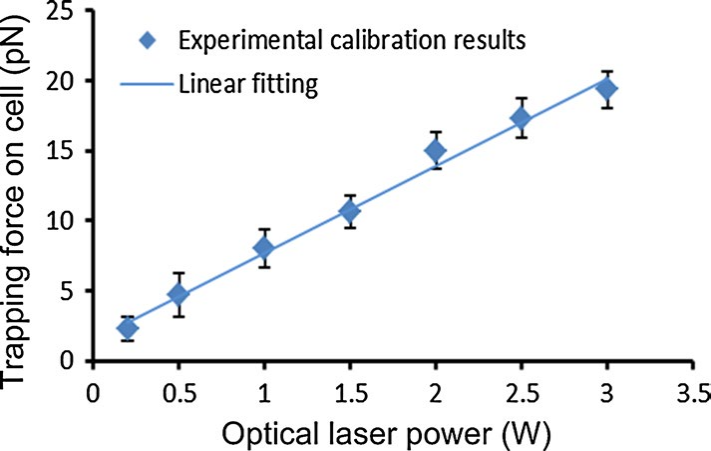
Calibration of optical trapping forces under different laser powers

Figure 1 shows the force calibration results of human leukemia cell line Molm13 over a range of laser powers. The trapping force increased almost linearly with the laser power. To characterize the adhesion properties, different trapping forces were used by changing the laser power to manipulate cells and characterize the cell adhesion states.

### Cell culture and materials

Leukemia cell line Molm13 and stromal cell line M210B4, commonly used model systems for leukemia cell-marrow interactions [22–24] (American Type Culture Collection, Manassas, VA, USA), were cultured at 37 °C in 5 % CO_2_ in a humidified incubator. Both cell lines were maintained in RPMI 1640 medium supplemented with 10 % (v/v) fetal bovine serum (FBS, Invitrogen). AMD3100, a widely used drug that can selectively antagonize the binding of SDF-1 to CXCR4 and preferentially mobilize leukemic blasts into the peripheral circulation, was chosen to treat leukemia cells. Polyclonal goat anti-VCAM-1 antibodies (Santa Cruz) were used in combination with donkey anti-goat (Invitrogen) to mark VCAM-1 protein on leukemia cells. The SDF-1 protein expressed by stromal cells was stained with a rabbit polyclonal SDF-1 antibody (Santa Cruz) and goat anti-rabbit IgG-CFL 488 secondary antibody (Santa Cruz). The nucleus was visualized with DAPI.

### CXCR4 expression flow cytometry

For CXCR4 expression studies, leukemia cancer cell lines were adjusted to a density of 0.5 × 10^6^/ml in culture medium. Cells were washed with a 20-fold volume of ice-cold buffer without FBS, stained at 4 °C with saturating concentrations of phycoerythrin-conjugated anti-CXCR4 antibody (Life Technologies Corporation), and then analyzed by flow cytometry.

### Fluorescent staining confocal microscopy

Polyclonal goat anti-VCAM-1 antibodies (Santa Cruz) were used in combination with donkey anti-goat (Invitrogen) to mark VCAM-1 protein on leukemia cells. The SDF1 proteins expressed by stromal cells were stained with a rabbit polyclonal SDF1 antibody (Santa Cruz) and goat anti-rabbit IgG-CFL 488 secondary antibody (Santa Cruz). The nucleus was visualized with DAPI.

Cells were washed twice with 1 × PBS and fixed in 3.7 % formaldehyde for 10 min at room temperature. The cells were then washed three times and permeabilized with 0.5 % Triton X-100 in PBS. After 5 min, cells were washed again and blocked with 5 % goat serum in PBS for 20–30 min. Cells were incubated with antibody for 1 h at 37 °C, washed three times with PBS, and incubated for 45 min at 37 °C with secondary antibody. Cell nucleuses were stained with DAPI for 5 min at room temperature. The cells were then washed three more times and observed under a laser-scanning confocal microscope (Leica microsystem, Wetzlar, Germany).

### Retrograde flow assay

The dynamics of the retrograde flow in stromal cells lamellipodia was characterized by tracking the motion of microparticles on cell leading edge. The microparticles were prepared as reported [25], and positioned by optical tweezers to adhere on the stromal cell leading edge. Optical tweezers was then switched off, and the position of the microparticle was measured over a time course of 5 min. The retrograde transport velocity of the microparticle was analyzed by image processing.

### Data analysis

Data were represented by the mean value ± standard error mean. The statistical differences or similarities between the groups were studied using t test. Groups were considered to have significant difference with p values lower than 0.05.

## Experiments and results

### Operation principle

**Fig. 2:**
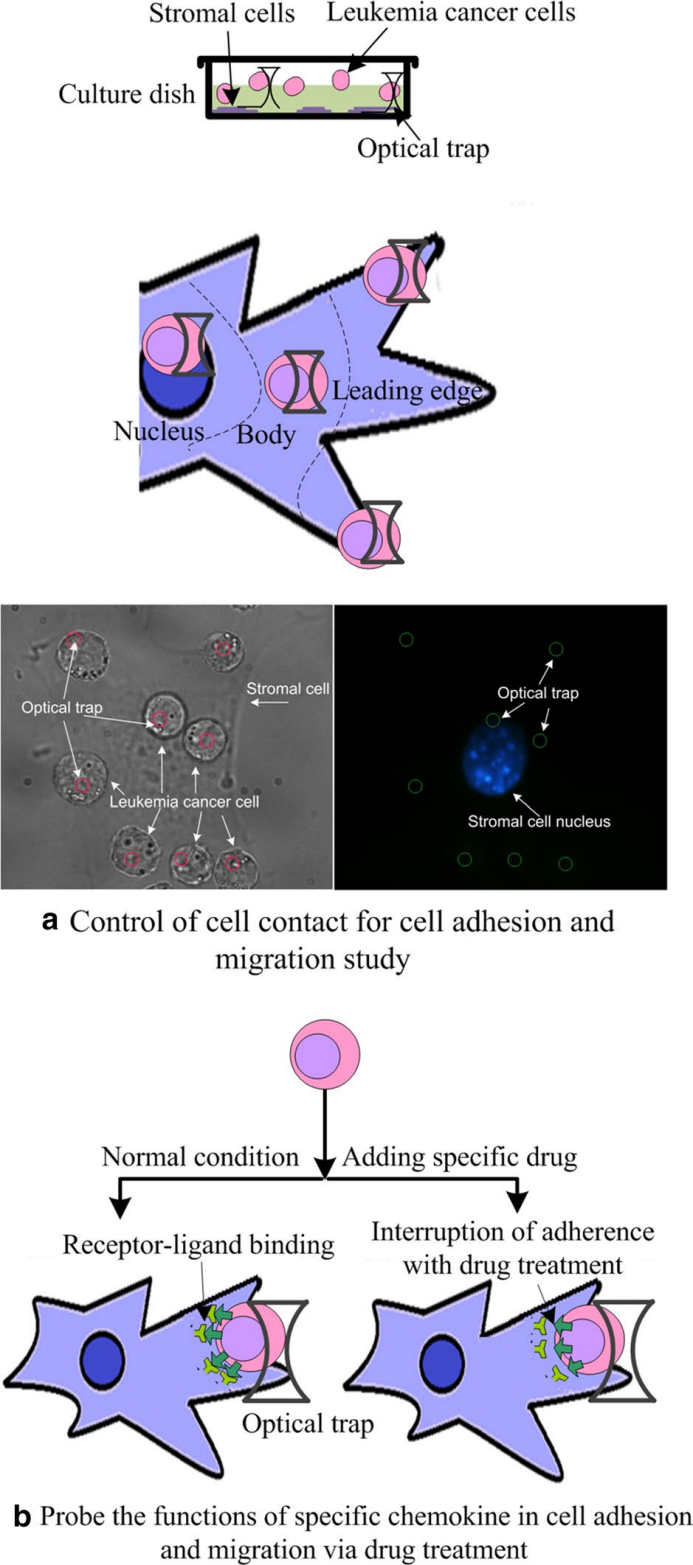
Operation principle. **a** Control of cell contact for characterizing cell adhesion and migration. Leukemia cells were assembled in three different locations of stromal cells, namely, nucleus, cell body, and cell leading edge. **b** Regulating cell-to-cell interactions via drug treatment

Figure 2 illustrates the operation principle of controlling cell contact sites for initial cell-to-cell interaction study. As shown in Fig. 2a, optical tweezers were used to place one type of cells (i.e., leukemia cancer cells) and assemble them at varied distances with respect to the nucleus of the other type of cells (i.e., stromal cells). The optical tweezers employed small laser power (i.e., 50 mW, corresponding to a trapping force of about 500 fN) to maintain cell contact for a few minutes. The powers of these optical tweezers were then turned off, and the other single optical tweezers was used to move the cells to detect the adhesion states. Time lapse video was utilized to record the mobilization of each single cells. The position of each cell was analyzed based on image processing to help identify cell migration. This method enabled us to characterize the initial cell adhesion and cell migration at different contact sites.

The functions of specific chemokine in cell adhesion and migration were studied using drug to block a signaling pathway involved in these processes. Figure 2b shows the characterization of adhesion and migration via drug treatment. One type of cell (i.e., leukemia cancer cell) was pretreated with specific drug to selectively antagonize the binding of receptor-ligand and then co-cultured with the other type of cell (i.e., stromal cell) for 2 h. The adhesion and migration were then analyzed based on the proposed method.

A case study was performed to investigate the adhesion between leukemia cancer cells and bone marrow stromal cells. As reported in the literatures, SDF-1 and CXCR4 regulate the adhesion, homing, and mobilization of leukemia cells [26–28]. Disruption of these interactions by SDF-1/CXCR4 antagonists represents a novel strategy for targeting leukemia/bone marrow microenvironment interactions [29, 30]. In clinical applications, AMD3100 [30–32], a bicyclam molecule that selectively antagonizes the binding of SDF-1 to CXCR4, has been used to disrupt the interaction of leukemic cells with the marrow microenvironment and mobilize leukemic blasts from their protective microenvironment into the peripheral circulation, making them more accessible to in vivo cytotoxic chemotherapy. In this paper, the role of SDF-1/CXCR4 signaling pathway to the interaction between leukemia cells and bone marrow cells at single cell level was studied using the proposed cell adhesion manipulation tool.

### Characterization of adhesion property

**Fig. 3:**
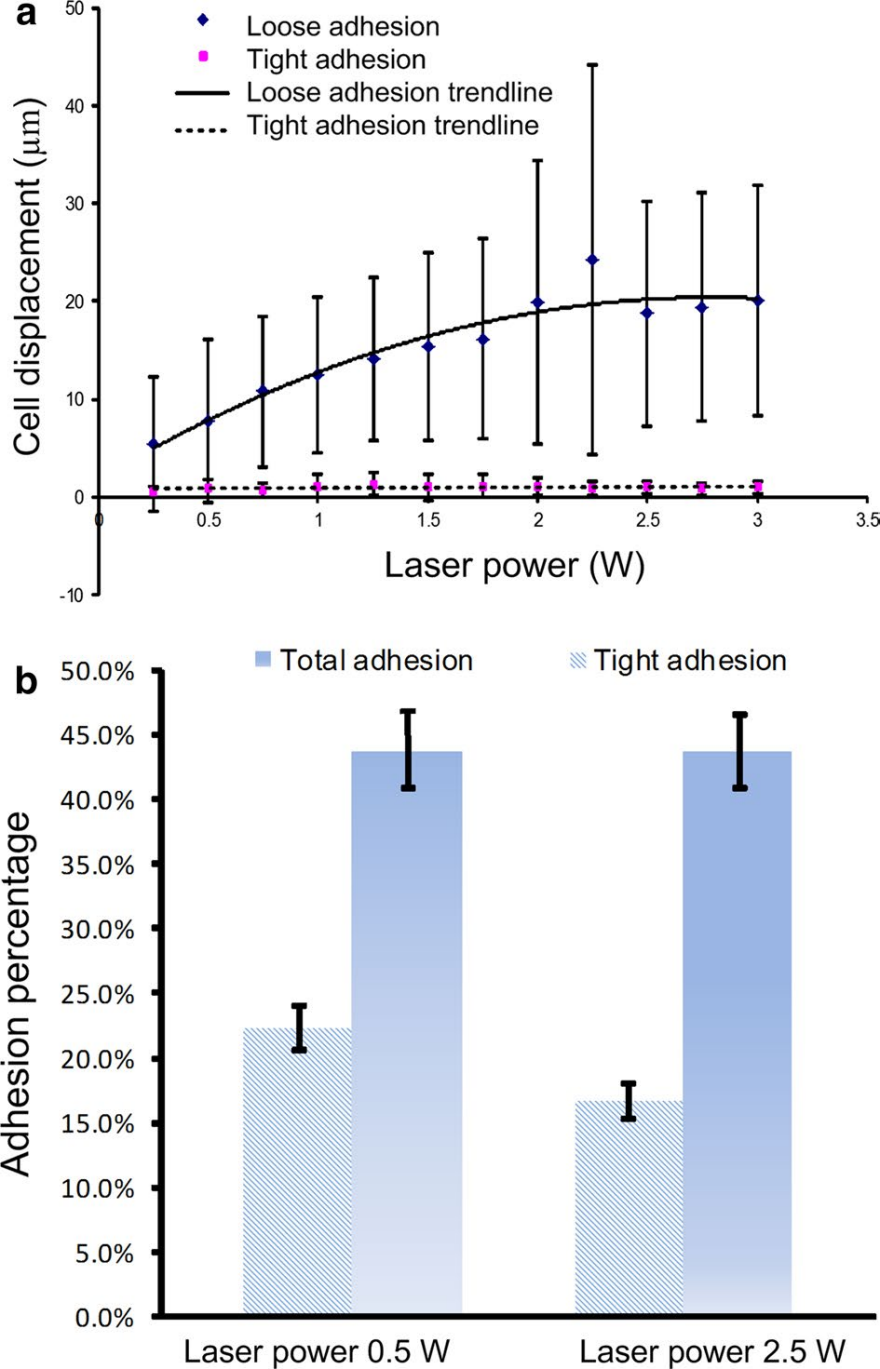
Adhesion states of leukemia cells. **a** Characterization of tightly and loosely adherent cells under varied laser powers. **b** Adhesion characterization of leukemia cells on stromal cells

We firstly used optical tweezers with different laser powers to move the leukemia cells to identify the state of adhesion of leukemia cells on stromal cells based on the ease of movement. The adhesion states were classified as tight adhesion, loose adhesion, and free suspending [16]. The “tight adhesion” cells could not be moved away when the laser power was increased from 0 to 3 W, corresponding to a trapping force of 0 to 20 pN (Fig. 3a). The “loose adhesion” cells could be moved away from their original sites. The moving displacement of these cells, defined as the largest distance that the leukemia cell can be moved away by optical tweezers with respect to its original adhesion site, increased as the laser power increased. The displacement increased moderately when the laser power exceeded 2.25 W. The maximum displacement appeared at the laser power around 2.25 W, which corresponded to a trapping force of 16 pN. When the laser power exceeded 2.5 W, the cells could be moved at least 5 µm from their original adhesion site. Cells that did not adhere on stromal cell layer were classified as “free suspending” cells.

To characterize the adhesion properties of cells, percentages of leukemia cancer cells adhered to stromal cells at the two laser powers of 0.5 and 2.5 W were examined. Figure 3b shows that 44 % of Molm13 cells adhered to M210B4 cells at the laser power of 0.5 W, whereas 23 % of these cells tightly adhered to stromal cells. At the laser power of 2.5 W, the adhesion percentage did not change markedly, but the number of tightly adherent cells was reduced to 18 %. These results indicate that a large trapping force can better distinguish the tightly and loosely adherent cells. Characterization of cell adhesion states can help identify cell adhesion ability after the cells are treated by drug or assembled at different sites.

### Characterization of the influence of SDF-1/CXCR4 signaling pathway in cell adhesion

**Fig. 4:**
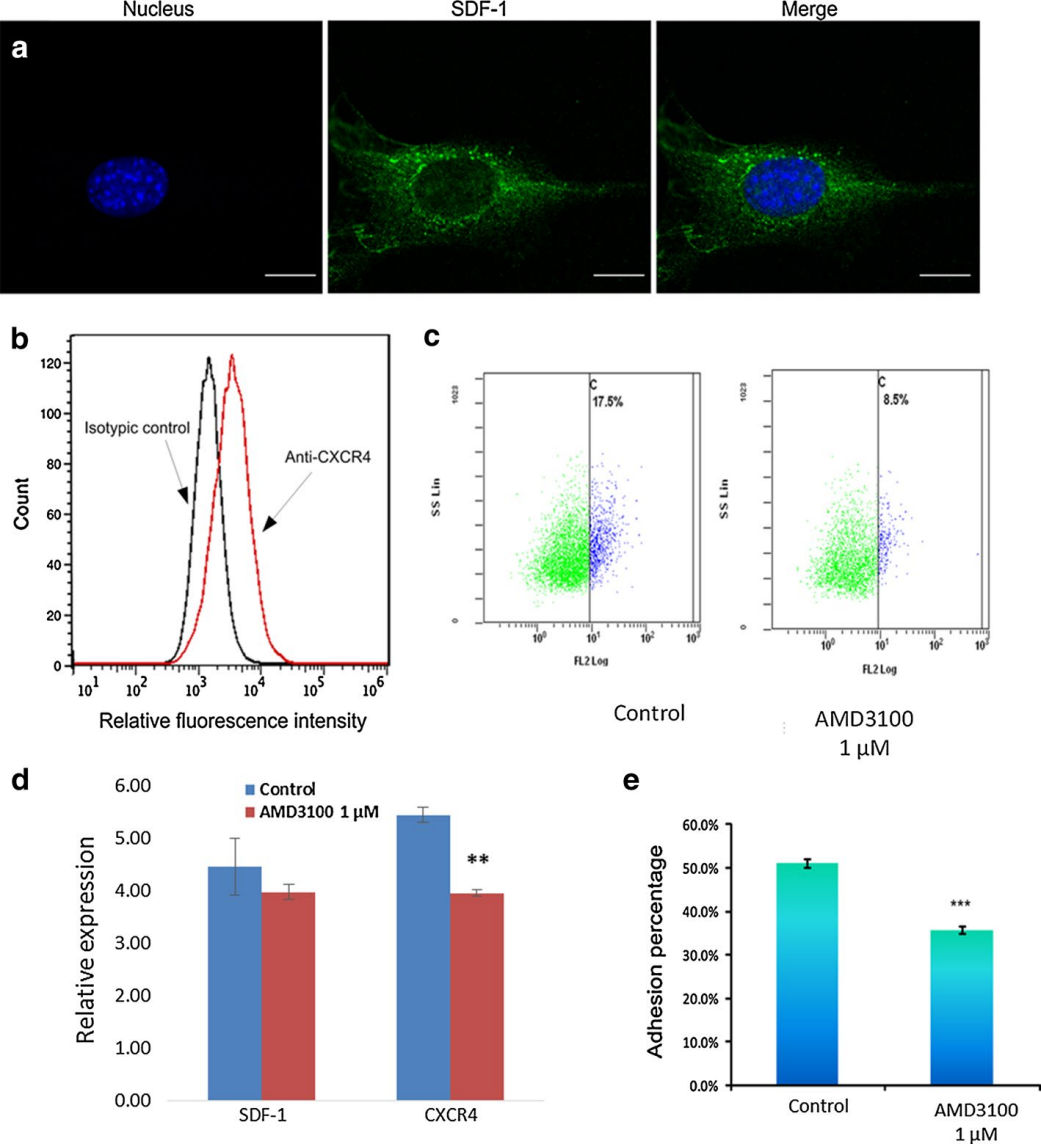
**a** 3D confocal images of SDF-1 staining on M210B4 stromal cells in X–Y plane. The *scale bar* is 10 µm. **b** Surface expression of CXCR4 on Molm13 cells. Molm13 cells were incubated with anti-CXCR4 and labeled with an FITC conjugate. Controls received equivalent concentrations of isotype-matched IgG. Washed cells were analyzed by flow cytometry, in which accumulated events were gated against the isotype control. **c** Flow cytometer tested the expression of CXC4 in molm13 cells. The CXCR4 expression cells decreased after 1 μM AMD3100 treated. **d** Expression of *sdf*-*1has* no significant difference after 1 μM AMD3100 treated in M210B4 cells. *Cxcr4* expression significantly decreased after AMD3100 treated (p < 0.05). **e** Characterization of adhesion of Molm13 cells on M210B4 cells with and without drug treatment. Molm13 cells were treated with 1 µM AMD3100 for 2 h before experiments. Experiments were repeated for eight times. *P < 0.05. ***P < 0.001

We then verified whether blocking the SDF-1/CXCR4 signaling pathway could influence the adhesion between leukemia and stromal cells. According to the literatures, SDF-1 is one of the most important chemokines that can induce adhesion and migration by inducing integrin activation [33–35]. In our study, we analyzed the SDF-1 protein on M210B4 cells by fluorescence staining. M210B4 cells express high levels of SDF-1 protein and can stimulate the adhesion and migration of CXCR4-expressing cancer cells [36]. Figure 4a illustrates 3D confocal microscopy images of SDF-1 staining on M210B4 stromal cells in the X–Y plane, showing that the SDF-1 molecules are distributed in the whole stromal cell. The fluorescence of the stromal cell exhibited high intensity near the cell nucleus, and low intensity on the nucleus and the leading edge of the cell. We also analyzed CXCR4 expression in Molm13 cells, as seen in Fig. 4b. It is seen that the fluorescence intensity of the cells treated with anti-CXCR4 had a shift compared to that of isotype control cells, indicating that Molm13 cells expressed CXCR4 molecules on the cell membrane.

We further characterized the influence of SDF-1/CXCR4 signaling pathway on adhesion of Molm13 cells to M210B4 stromal cells, using AMD3100 as an inhibitor to inhibit CXCR4 expression. Molm13 cells were pre-incubated with 1 µM AMD3100 for 2 h before the adhesion assay. Figure 4c, d illustrate that the CXCR4 expression significantly decreased in mRNA and protein level after AMD 3100 treatment. Figure 4e illustrates the percentage of Molm13 cells adhered on M210B4 cells with and without AMD3100 drug treatment, respectively, wherein both tightly and loosely adherent cells were classified as adherent cells with manipulation force of 16 pN. It is obvious that the number of adherent Molm13 cells decreased after the pretreatment with 1 µM AMD3100, which was in agreement with the results of other studies [9, 37]. All these results indicated that blocking the signaling pathway of SDF-1/CXCR4 through drug treatment could successfully affect the adhesion ability of leukemia cells on stromal cells.

### Control of adhesion contact sites

**Fig. 5:**
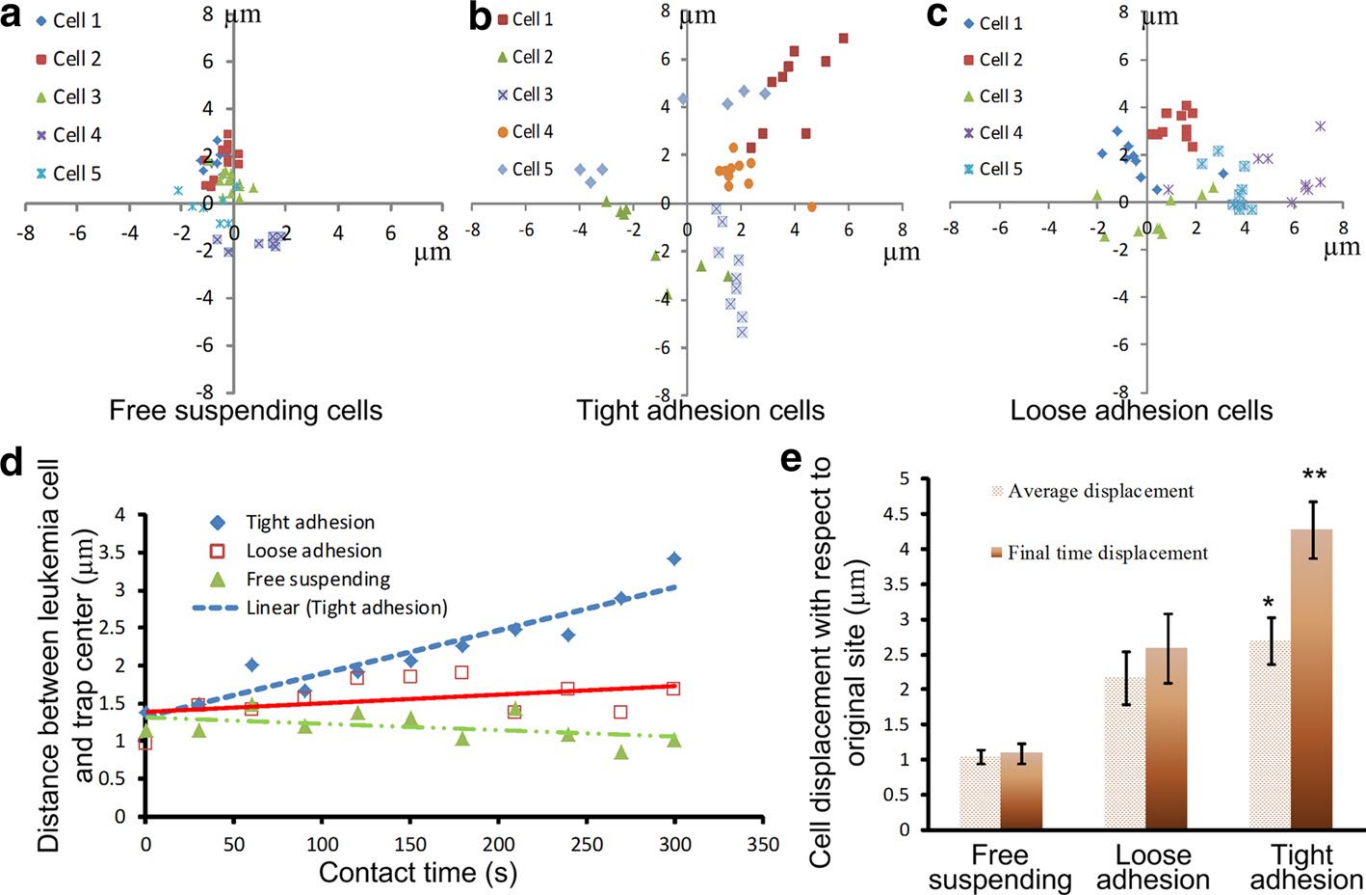
Controlled cell adhesion contact with optical tweezers. **a**–**d** Analysis of distance between leukemia cell and trap center. **e** Leukemia cell displacements with respect to the initial contact sites. *P < 0.05. **P < 0.01

We further analyzed how adhesion contact site affects the cell adhesion and interaction. In our experiment, the Molm13 leukemia cells were assembled on the M210B4 stromal cell layer, and their contacts were maintained by exerting small trapping forces of 500 fN on the leukemia cells for 5 min. Figure 5a–d illustrate displacements of the leukemia cells with respect to the trapping center of the optical tweezers. The free-suspending cells appeared small deviations from the trapping center because of Brownian motion. The tightly adherent cells slightly moved away from the trapping center, indicating a directed migration that may be induced by the activation of the SDF-1/CXCR4 signaling pathway. The loosely adherent cells exhibited the moving performance between the free-suspending and tightly adherent cells. These results indicated that small trapping forces acting on Molm13 cells could successfully maintain the cell at the initial contact site before the cell tightly adhered to the stromal cell. The displacements of leukemia cells with respect to their original positions were also quantitatively analyzed. Figure 5e illustrates the average displacement of the Molm13 cells based on the measured data during the initial 5 min (data were acquired every 30 s), together with the final displacement measured at 5 min. For the free suspending cells, the final displacement and the average displacement were almost the same, indicating that directed migration did not happen. For the tightly adherent cells, the final displacement was larger than the average displacement, implying that the tightly adherent cells migrated away from the trapping center. For the loosely adherent cells, the final displacement was different from the average displacement, but the difference was not as large as the tightly adherent cells. These results demonstrated that the migration ability of leukemia cells on stromal cell layer could be affected by the adhesion state, and the tightly adherent cells appeared to have more chance to migrate.

**Fig. 6:**
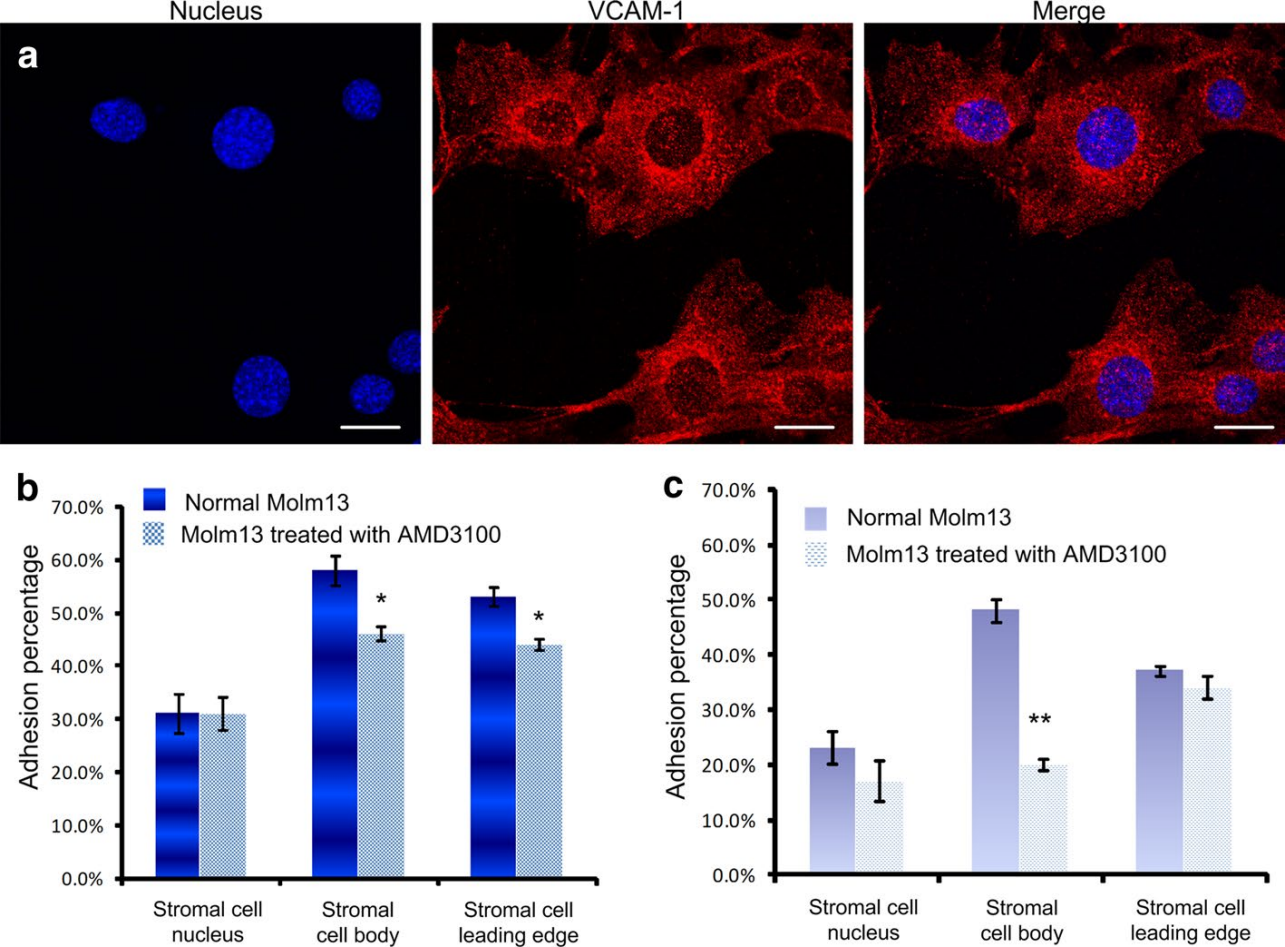
**a** Confocal images of VCAM-1 staining on M210B4 stromal cells. The *scale bar* is 20 µm. **b** Total adhesion percentage of Molm13 cells assembled at different positions of M210B4 cells. Two groups of Molm13 cells, namely, the normal group and group treated with 1 µM AMD3100 for 2 h before experiments. **c** Tight adhesion percentage of Molm13 cells assembled at different positions of M210B4 cells. Two groups of Molm13 cells, namely, the normal group and group treated with 1 µM AMD3100 for 2 h before experiments were studied.*P < 0.05. **P < 0.01

We then examined the adhesion molecule distribution by staining vascular cell adhesion molecule-1 (VCAM-1) on the stromal cell surface with specific antibody and analyzing the confocal microscopy images. VCAM-1, which is constitutively expressed in bone marrow stromal cells, has been considered as a key protein that regulates the adhesion of α_4_β_1_-integrin (the receptor for VCAM-1) expressed by leukemia cells [38–40]. Figure 6a shows that the VCAM-1 adhesion molecules were distributed mainly on the stromal cell body and the cell leading edge, and a small amount of VCAM-1 protein was found on the membrane around the cell nucleus.

We finally studied the influence of contact sites on the cell adhesion property. The nucleus of stromal cell M210B4 was stained with DAPI, and the distances between leukemia and stromal cells were calculated by image processing. The leukemia cells were assembled in three different locations of the stromal cells, namely, nucleus, cell body, and cell leading edge, with the distance between the leukemia cell center and stromal cell nucleus center of 8 ± 1, 19 ± 1, and 35 ± 4 µm, respectively. For the group of cells assembled on the stromal cell nucleus, about 31 ± 11 % Molm13 cells adhered to the M210B4 stromal cell layer (Fig. 6b), where 23 ± 9 % Molm13 cells tightly adhered on stromal cells (N = 3, n = 59) (Fig. 6c). For the group of cells adhering on the stromal cell body, 58 ± 9 % Molm13 cells adhered to the stromal cell layer, where about 48 ± 6 % cells tightly adhered (N = 3, n = 55). For the group of cells adhering on the stromal cell leading edge, the percentage of adhesive cells reached 53 ± 5, and 35 ± 3 % tightly adhered on stromal cells (N = 3, n = 78).

Furthermore, Molm13 cells pretreated with 1 µM AMD3100 for 2 h were assembled on the stromal cell layer. Figure 6b shows that the adhesion property of cells, before and after drug treatment, did not change obviously when the Molm13 cells were placed near the stromal cell nucleus (N = 3, n = 69). When the Molm13 cells were placed on the stromal cell body (N = 3, n = 44) or leading edge (N = 3, n = 93), the adhesion percentages of Molm13 cells were decreased. These results implied that AMD3100 could reduce adhesion ability of the Molm13 cells on the stromal cell, and the influence could be more significant when the Molm13 cells were placed on the stromal cell body and leading edge where the amount of VCAM-1 appeared to be higher. We therefore hypothesize that the amount and distribution of adhesion molecules could largely affect the cell-to-cell adhesion.

### Migration of leukemia cell on stromal cell layer

**Fig. 7:**
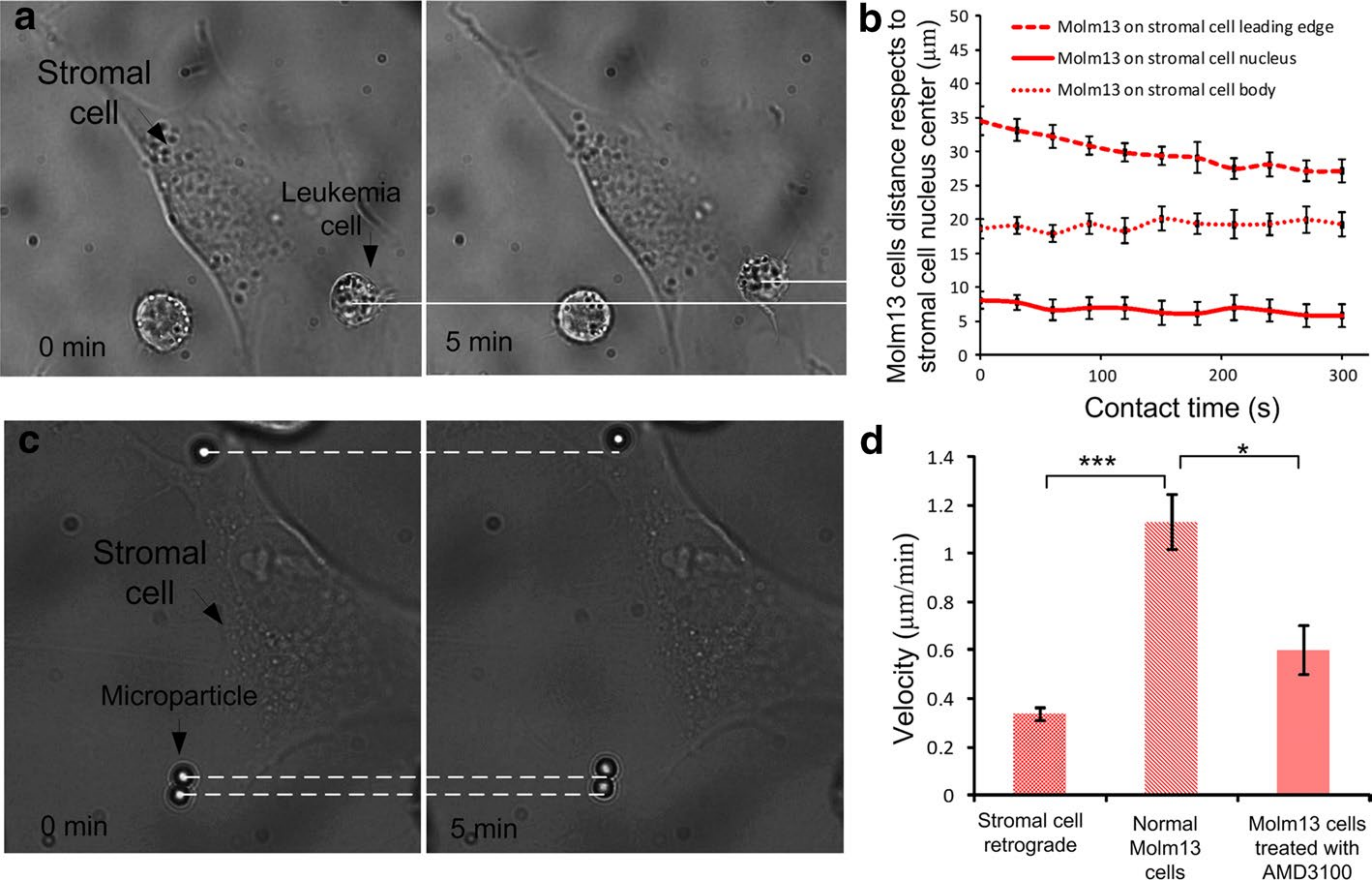
**a** Experimental images show that Molm13 cells migrate on the M210B4 cell leading edge toward the stromal cell nucleus. **b** Distance of Molm13 cells with respect to the nucleus center of M210B4 cells under tight adhesion. **c** Experimental images show the retrograde flow of M210B4 cells. **d** Velocities of retrograde flow of M210B4 cells and migration velocity of Molm13 cells on M210B4 with or without AMD3100 treatment

It has been reported that the SDF-1 chemokine, which binds to CXCR4, regulates the trafficking of CXCR4+ leukemia cells [41, 42]. To verify whether the SDF-1 secreted by M210B4 bone marrow stromal cell caused the directed migration of CXCR4-expressed Molm13 cells, the relative motion between tightly adherent leukemia cells and stromal cells was examined. Figure 7B illustrates the distance between the leukemia cell center and the stromal cell center, indicating that the Molm13 cells on the stromal cell leading edge experienced directed migration toward the stromal cell nucleus. However, the Molm13 cells on the stromal cell body and nucleus did not show the directed migration, which agreed to the expression of SDF-1 in Fig. 4a (where the SDF-1 had the highest expression on the stromal cell body). A gradient field from stromal cell body to stromal cell leading edge could be the reason why the leukemia cells on the stromal cell leading edge could migrate to the stromal cell body.

We also investigated that the migration of leukemia cells on the stromal cell leading edge was caused by the remodeling and shape changing of the stromal cell [14] but not the retrograde flow of the stromal cell. To verify this, we examined the motions of the stromal cell and leukemia cells quantitatively. The velocity of stromal cell nucleus was measured to be 0.44 ± 0.06 µm/min, and the retrograde velocity of stromal cell leading edge was 0.34 ± 0.02 µm/min. The velocity of leukemia cell moving on the stromal cell leading edge was 1.19 ± 0.09 µm/min, which was much higher than the retrograde flow of stromal cell. This verified the SDF-1 induced cell migration. We further examined the migration of Molm13 cells after pretreatment with AMD3100. According to the literature, AMD3100 could block the signaling pathway [31]. Our results in Fig. 7D showed that the migration velocity of leukemia cells decreased to 0.62 ± 0.08 µm/min after treatment, indicating that AMD3100 weakened the migratory ability of leukemia cells.

## Discussions

Understanding the cell-to-cell interaction process, specifically the cell adhesion and migration involved in a particular signaling pathway, is important to characterize cell functions and develop novel strategy for target therapy. Some new technologies based on unique tools such as AFM, microfluidic technology, and optical tweezers have been developed to study the cell-to-cell interaction process at single cell level; however, an efficient method that can manipulate and control single cells for probing cell adhesion and migration in a special signaling pathway remains elusive. This paper presented a new method to control cell adhesion with optical tweezers for investigating the functions of specific signaling pathway involved in cell-to-cell interaction at single cell level.

The holographic optical tweezers system allows the manipulation of cells with trapping force ranged from several picoNetwon to tens of picoNewton. Previous studies have shown that the receptor-ligand binding force is about hundreds of picoNewtons [43, 44], indicating that the trapping force in our system would not break the receptor-ligand binding and hence is safe to characterize cell adhesion states. This was verified in this study by characterizing the cell adhesion percentage of Molm13 cells on M210B4 cells with different manipulation forces. The experimental results have evidenced that the adhesion percentage did not markedly change (Fig. 3b) when the trapping force was ranged from 4 to 16 pN. We also evaluated the cell adhesion property under different manipulation forces, and our data indicated that the Molm13 cells adhering on M210B4 could be classified in two categories, the loose adhesion and the tight adhesion cells (Fig. 3a). This classification could help evaluating the ability of the cell adhesion and cell migration. Notably, the tight adhesion cells exhibit more obvious directed migration compared with the loosely adherent cells (Fig. 5).

The SDF-1/CXCR4 signaling pathway regulates leukemia cell adhesion and migration. Receptor-ligand SDF-1/CXCR4 affects downstream ligand molecule in bone marrow microenvironment, and also affects the tumor growth through adhesion [33, 45]. However, the observation of cell adhesion and migration involved in this signaling pathway at single cell level is lacking. Our experimental results (Fig. 4c, d) demonstrated that the drug AMD3100 could downregulate the CXCR4 expression in leukemia cell and influence the adhesion and migration of leukemia cells. Our analysis of the adhesion percentage showed that 1 µM AMD3100 could significantly reduce the adhesion between Moml13 cells and M210B4 cells. The analysis of initial adhesion by controlling the cell contact site through optical tweezers manipulation showed that the effect of AMD3100 was more obvious on stromal cell body and leading edge, where the expression of the adhesion molecule was higher (Fig. 6). These findings are consistent with some existing studies, indicating that the ligand distribution and amount could largely affect the adhesion [46, 47].

By assembling leukemia cells on stromal cell layer and maintaining the cell position with a tiny trapping force, we found that the tightly adherent cells exhibited directed migration on stromal cell layer (Fig. 5), especially on stromal cell leading edge (Fig. 7). This agreed to the literatures that the SDF-1 secreted by bone marrow cells induces and regulates cell migration and traditional cell migration assay [10, 37, 40, 41]. To exclude the influence of the retrograde flow on cell migration, we investigated the retrograde flow of M210B4 cells quantitatively by assembling specific functionalized beads on stromal cell layer. A similar study was performed in [12]. Our data suggested that the retrograde flow velocity of the M210B4 was lower than the migration velocity of leukemia cell on the stromal cell leading edge, verifying the SDF-1 induced cell migration. We further showed that pretreatment with AMD3100 weakened the migration of leukemia cells (Fig. 7).

## Conclusions

This paper reported a new method to study specific signaling pathway in cell-to-cell interaction at single cell level, through cell adhesion control with optical tweezers. We successfully characterized the property of adhesion between leukemia cancer cells and bone marrow stromal cells quantitatively, and analyzed the SDF-1/CXCR4 interaction with drug treatment. Control of the adhesion of leukemia cells on the stromal cell layer showed that the amount of adhesion molecules largely affected cell-to-cell adhesion. We also found that leukemia cells could be induced to migrate to the stromal cell layer under the stimulation of SDF-1. The proposed method provides a new and efficient way to observe the role of SDF-1/CXCR4 involved in cell-to-cell interaction initially. In the future research, we will continue to probe the mechanism of how SDF-1/CXCR4 signaling pathway affects the cell–cell interactions.

## Abbreviations

AFM: atomic force microscopy
SDF-1: stromal-derived factor 1
VCAM-1: vascular cell adhesion molecule-1
